# Supplementary material for: Aggregation-regulated room-temperature phosphorescence materials with multi-mode emission, adjustable excitation-dependence and visible-light excitation
Source: Nat Commun. 2023 Jul 13;14:4163. doi: 10.1038/s41467-023-39767-w (PMC10344924; doi:10.1038/s41467-023-39767-w)
Supplement: Supplementary file 1 — Supplementary Information [file 41467_2023_39767_MOESM1_ESM.pdf]

Supplementary Information

Aggregation-Regulated Room-Temperature Phosphorescence Materials with Multi-Modes Emission, Adjustable Excitation-Dependence and Visible-Light Excitation

You *et al.*

Supplementary Experimental section

Materials. Microcrystalline cellulose (MCC) with degree of polymerization (DP) of 280 was acquired from Beijing Fengli Jingqiu Commerce and Trade Company (China). The DP of MCC was determined by GPC-MALS in our previous work[29]. MCC was dried under vacuum at 80 °C for 24 h. Cellophane was provided by Shandong Henglian New Materials Co., Ltd. 1-Allyl-3-methylimidazolium chloride (AmimCl) was synthesized in the laboratory. The water content in AmimCl determined by Karl Fischer method was less than 0.3 wt%. Trimellitic anhydride, 4-dimethylaminopyridine (DMAP), sodium bicarbonate ($NaHCO_3$), sodium hydroxide (NaOH), calcium chloride ($CaCl_2$), sodium carbonate (Na_2CO_3), barium chloride ($BaCl_2$), sodium sulfate (Na_2SO_4), aluminum chloride ($AlCl_3$), zinc chloride ($ZnCl_2$), indium chloride ($InCl_3$) and lanthanum chloride ($LaCl_3$) were purchased from Innochem and J&K Scientific. Ethanol and N,N′-dimethylformamide were received from Tianjin Concord Technology Co., Ltd. Concentrated hydrochloric acid was purchased from Sinopharm Chemical Reagent Co., Ltd. (China). The dialysis bag with a molecular weight cut-off of 3500 was purchased from Beijing Ruida Henghui Technology Development Co., Ltd. (China). Ultra-pure water (>18.2 $M\Omega \cdot cm^{-1}$) from the Millipore Milli-Q system was used in all experiments.

Characterization. ^{1}H-NMR spectra were acquired on a Bruker AV-400 NMR spectrometer (Bruker, USA) with 16 scans at room temperature in DMSO-d_6. A 20-μL aliquot of CF_3COOH-d_1 was added to shift the signals of the free hydrogens to the downfield. FTIR spectrum was recorded from 650 to 4000 cm^{-1} with 32 scans on a Nicolet 6700 FT-IR spectrometer (Thermo Fisher, USA). Fluorescence and phosphorescence spectra were recorded with a Hitachi F-7000 fluorescence spectrophotometer (Hitachi, Japan). The photoluminescence quantum yield, phosphorescence quantum yield and phosphorescence lifetime were measured on an Edinburgh FLS980 steady-state transient fluorescence spectrometer (Edinburgh, UK) with an integrating sphere and a microsecond flash lamp. In phosphorescence mode,

delay time = 0.1 ms; gate time = 2.0 ms. The phosphorescence lifetime was measured by multi-channel single photon technology. Fluorescence and phosphorescence images were captured with a digital camera (SONY α7, Japan). The state of CBtCOONa aqueous solution was measured on a dynamic light scattering instrument (ALV/CGS-3, Germany). Zeta potential of 1.0 mg/mL CBtCOONa aqueous solution was measured on Nano-ZS ZEN3600 (Malven Instruments, UK) at room temperature. The viscosity of CBtCOONa aqueous solution was measured on an AR2000ex rheometer (concentric cylinder) at 25 °C. SEM images of RTP samples were recorded with a S-4800 scanning electron microscope (Japan).

Density measurement of CBtCOONa film. CBtCOONa aqueous solution (3.0 mL, 200 mg/mL) was dropped onto glass plate. Then, the solvent was slowly evaporated at 60 °C for 2 h. The obtained film was dried under vacuum at 60 °C for 24 h. The dried CBtCOONa film was cut into square-shaped pieces. Their thickness, length and width were measured with micrometer to calculate the volume. Density was calculated by the formula $\rho = m/v$. This process was repeated four times.

Supplementary Figures and Tables

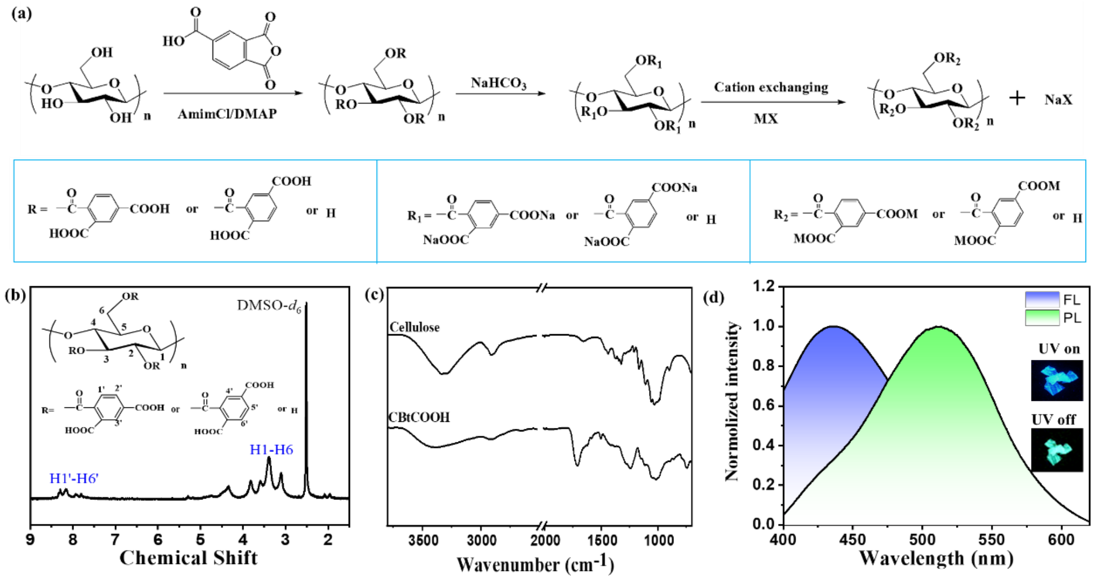

Supplementary Figure 1 Synthesis and characterization of CBtCOONa. (a) Synthetic route of CBtCOONa; (b) ^{1}H-NMR spectra of CBtCOONa (DS = 0.54); (c) FTIR spectra of CBtCOONa; (d) Normalized fluorescence and phosphorescence spectra of CBtCOONa (DS = 0.54) (Ex = 365 nm; delay time for phosphorescence spectrum = 0.1 ms) and photographs of CBtCOONa taken under 365 nm lamp and with the lamp off.

Supplementary Table 1 Synthesis of CBtCOOH under different reaction conditions.

Sample	Molar ratio of trimellitic anhydride and AGU	Molar ratio of DMAP and AGU	Reaction time/h	Temperature/°C	DS
1	2:1	0.1:1	12	80	0.33
2	2.5:1	0.1:1	12	80	0.54
3	3:1	0.1:1	9	80	0.63
4	3:1	0.1:1	12	80	1.12

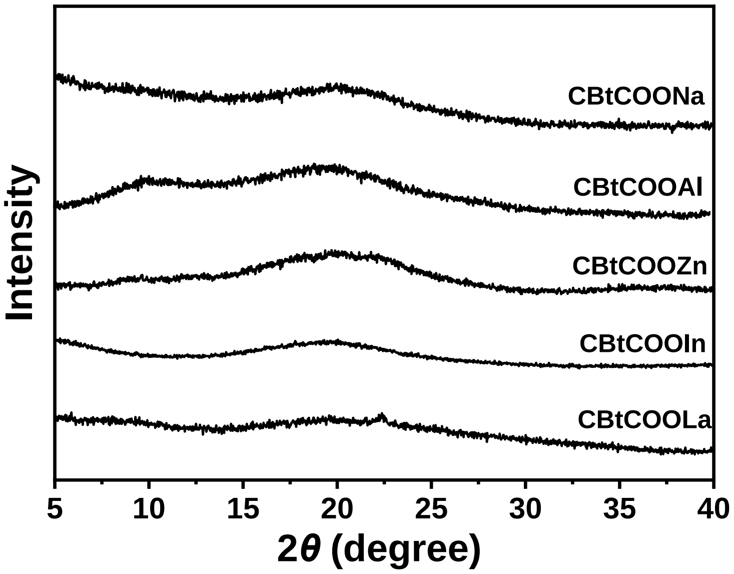

Supplementary Figure 2 XRD spectra of CBtCOOM.

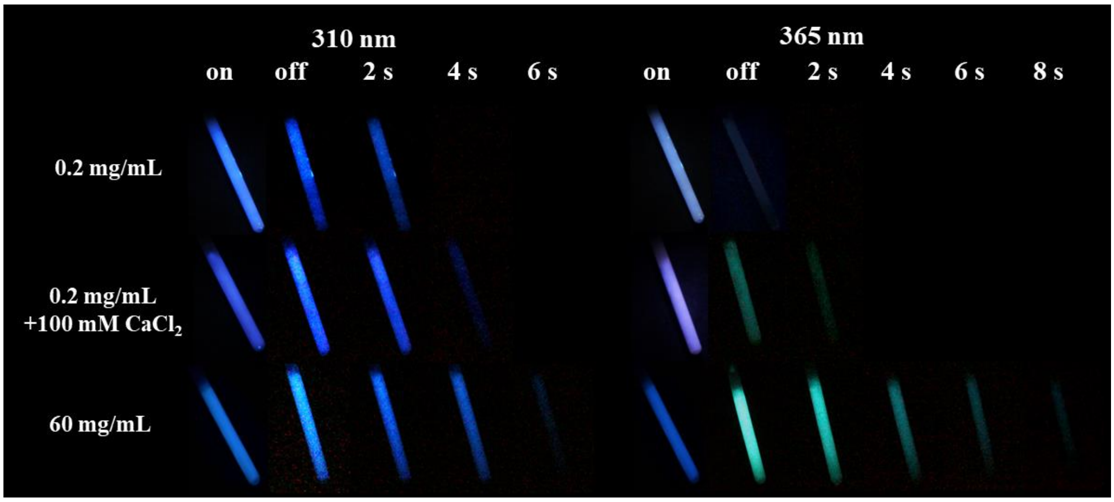

Supplementary Figure 3 Photographs of different concentrations of CBtCOONa aqueous solution at 77 K taken under 310 nm and 365 nm lamps and with the lamps off, respectively.

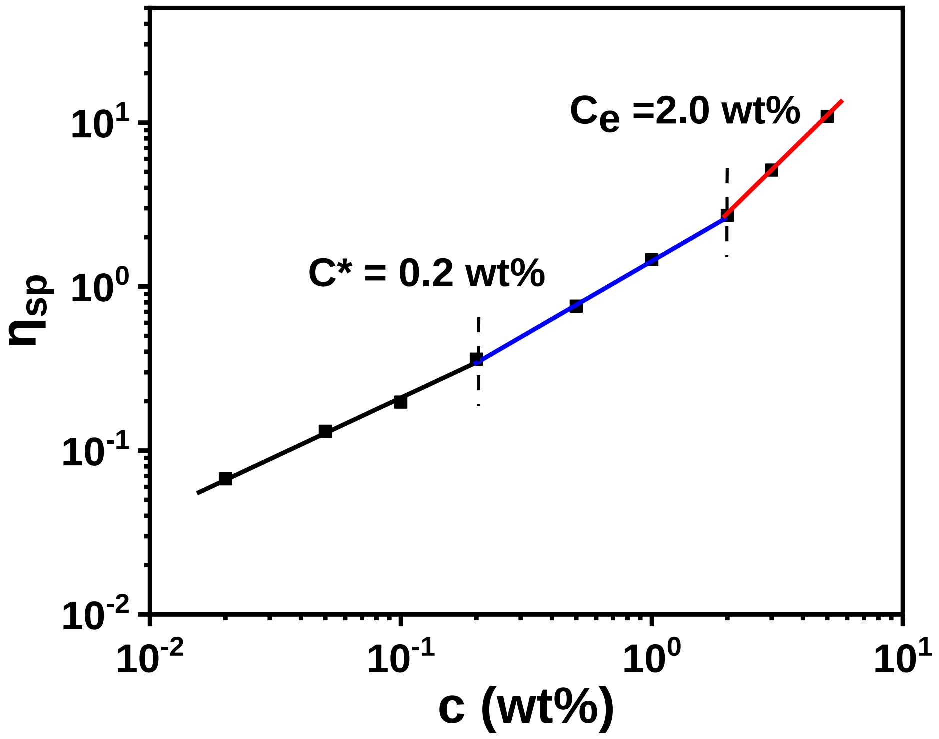

Supplementary Figure 4 Plot of η_{sp} versus the concentration of CBtCOONa solutions.

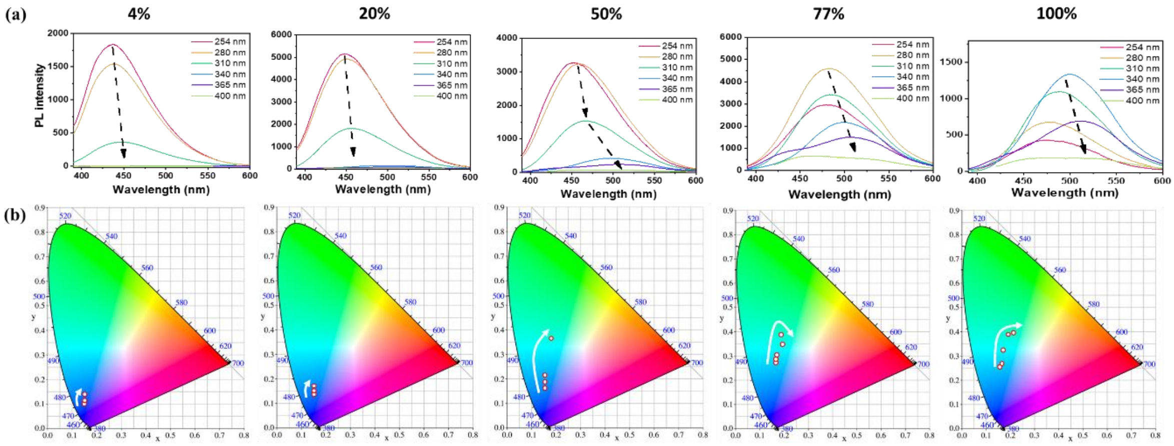

Supplementary Figure 5 (a) Phosphorescence spectra and (b) CIE coordinate diagrams of samples with different content of CBtCOONa.

Supplementary Table 2 CIE coordinate data of samples with different content of CBtCOONa.

CBtCOONa	254 nm	280 nm	310 nm	340nm	365 nm
4%	0.151,0.1047	0.1513,0.1112	0.1512,0.1365	-	-
20%	0.1502,0.1416	0.1507,0.1495	0.1508,0.1682	0.1806,0.3599	0.1930,0.3574
50%	0.1522,0.1621	0.1537,0.1839	0.1552,0.2148	0.1794,0.367	0.1920,0.3517
70%	0.1645,0.2731	0.1652,0.285	0.167,0.3025	0.1839,0.3876	0.1919,0.3496
100%	0.1576,0.2569	0.1626,0.2672	0.168,0.3248	0.1899,0.3882	0.2087,0.3944

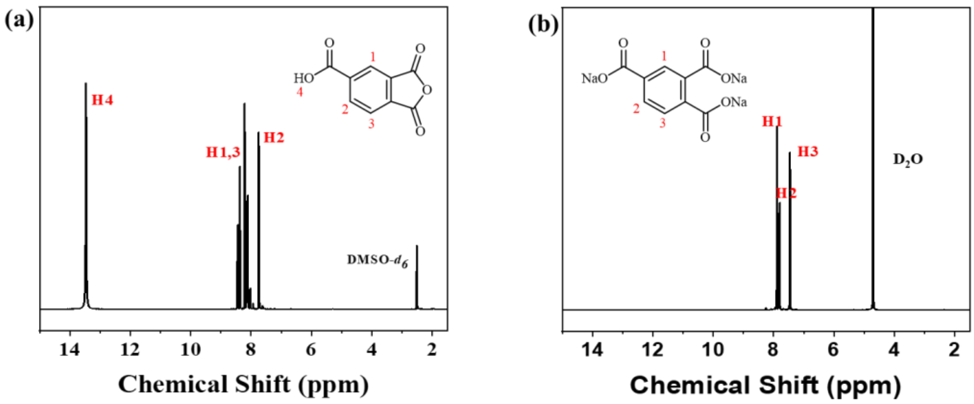

Supplementary Figure 6 ^{1}H-NMR spectra of (a) BtCOOH and (b) BtCOONa.

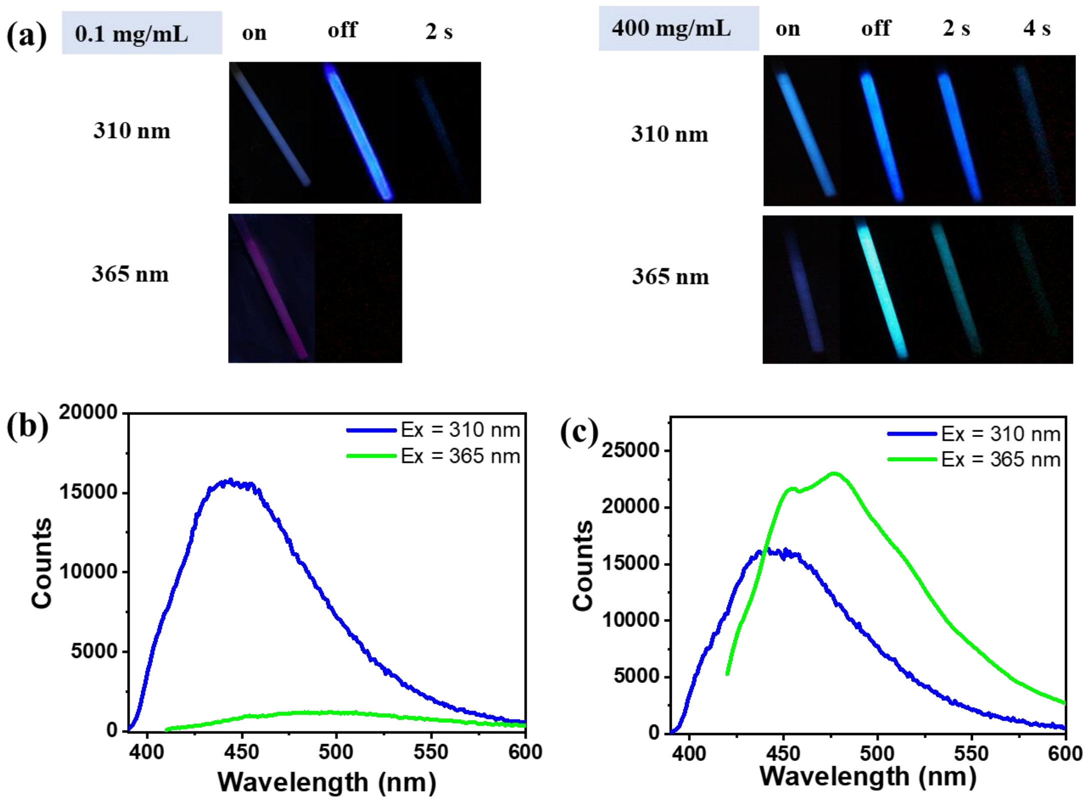

Supplementary Figure 7 (a) Photographs of BtCOONa with different concentrations at 77 K taken under 310 nm and 365 nm lamps and with the lamps off; (b) Phosphorescence spectra of 0.1 mg/mL BtCOONa at 77 K (EX = 310 nm and 365 nm; delay time = 0.1 ms); (c) Phosphorescence spectra of 400 mg/mL BtCOONa at 77 K (EX = 310 nm and 365 nm; delay time = 0.1 ms).

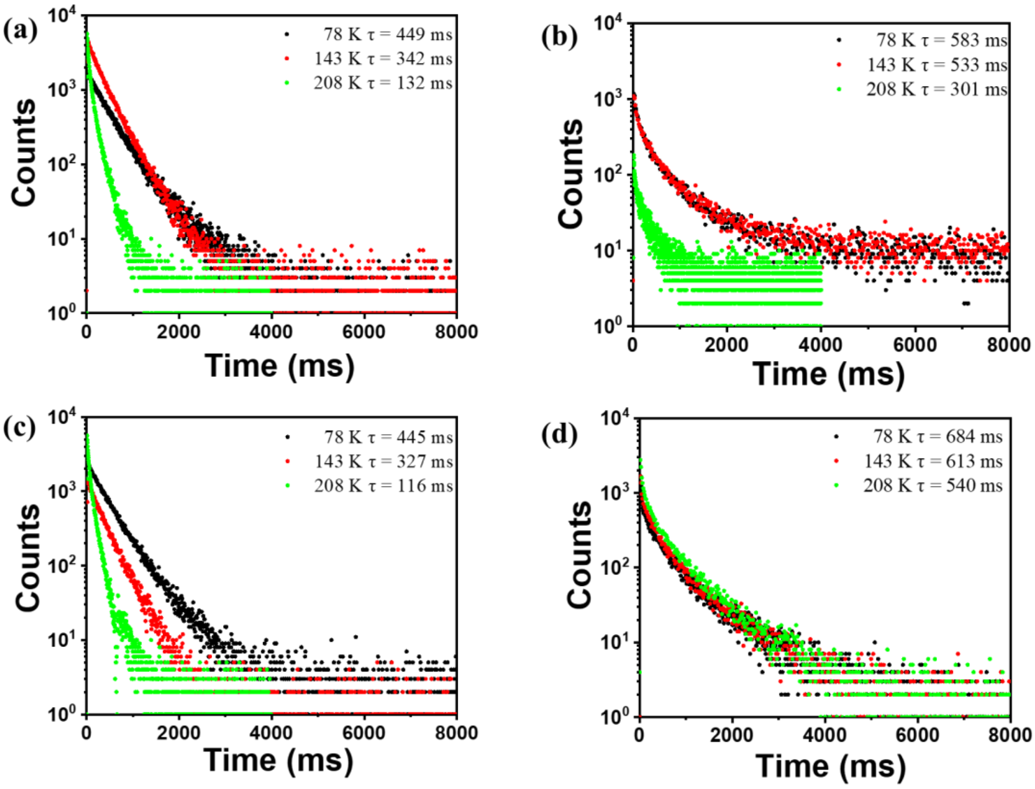

Supplementary Figure 8 (a) Phosphorescence lifetime spectra of CBtCOONa aqueous solution (0.1 mg/mL) at different temperatures (Ex = 310 nm; detection wavelength = 450 nm); (b) Phosphorescence lifetime spectra of CBtCOONa aqueous solution (0.1 mg/mL) at different temperatures (Ex = 370 nm; detection wavelength = 500 nm); (c) Phosphorescence lifetime spectra of CBtCOONa aqueous solution (400 mg/mL) at different temperatures (Ex = 310 nm; detection wavelength = 450 nm); (d) Phosphorescence lifetime spectra of CBtCOONa aqueous solution (0.1 mg/mL) at different temperatures (Ex = 370 nm; detection wavelength = 500 nm).

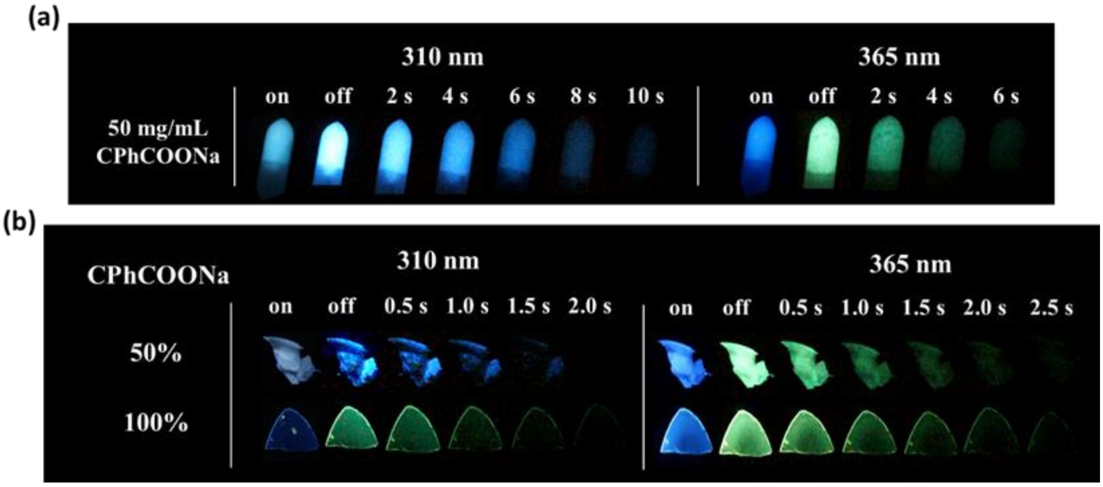

Supplementary Figure 9 (a) Photographs of 50 mg/mL CPhCOONa aqueous solution at 77 K taken under 310 nm and 365 nm lamps and with the lamps off, respectively. (b) Photographs of RTP

materials with 50% and 100% CPhCOONa contents under irradiation with 310 nm and 365 nm lamps and with the lamps off.

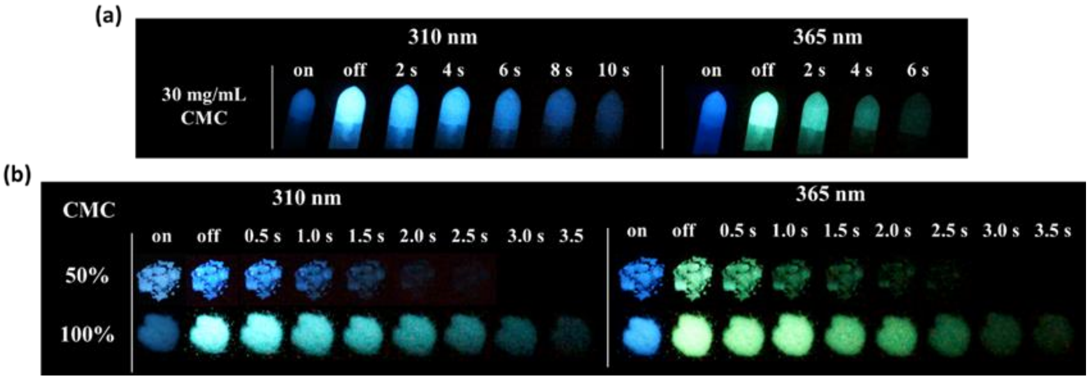

Supplementary Figure 10 (a) Photographs of 30 mg/mL CMC aqueous solution at 77 K taken under 310 nm and 365 nm lamps and with the lamps off, respectively. (b) Photographs of RTP materials with 50% and 100% CMC contents under irradiation with 310 nm and 365 nm lamps and with the lamps off.

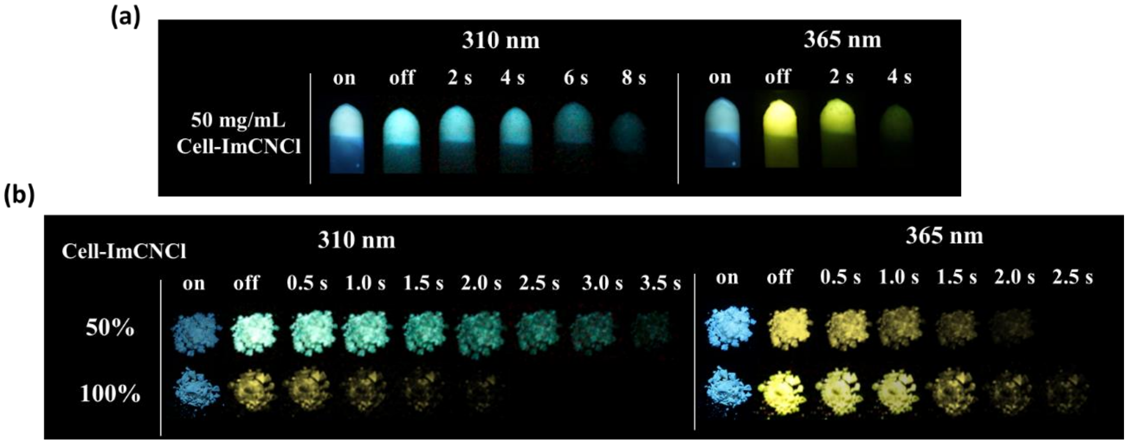

Supplementary Figure 11 (a) Photographs of 50 mg/mL Cell-ImCNCl aqueous solution at 77 K taken under 310 nm and 365 nm lamps and with the lamps off, respectively. (b) Photographs of RTP materials with 50% and 100% Cell-ImCNCl contents under irradiation with 310 nm and 365 nm lamps and with the lamps off.

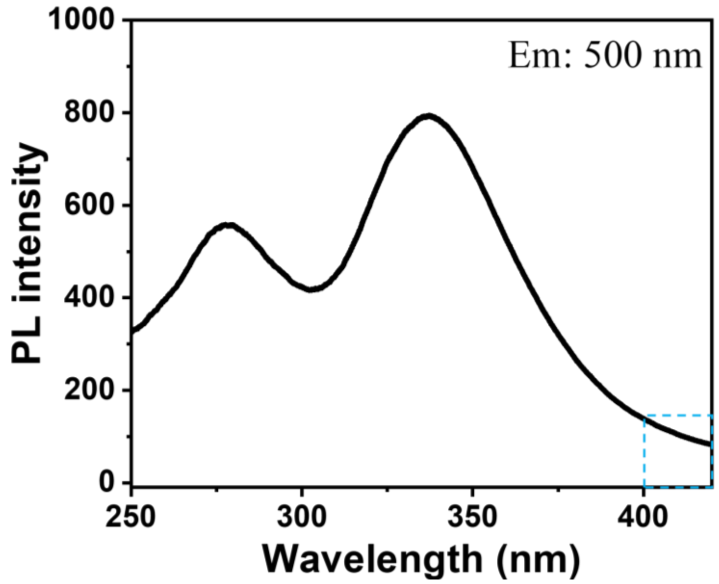

Supplementary Figure 12 Phosphorescence excitation spectra of CBtCOONa solid powder (Em = 500 nm).

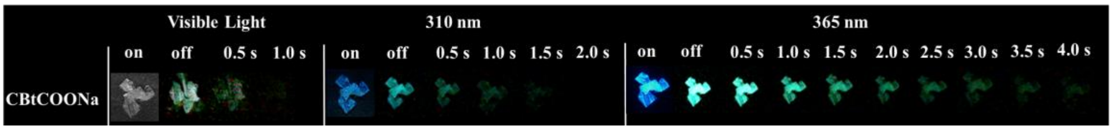

Supplementary Figure 13 Photographs of CBtCOONa taken under different lamps and with lamps off.

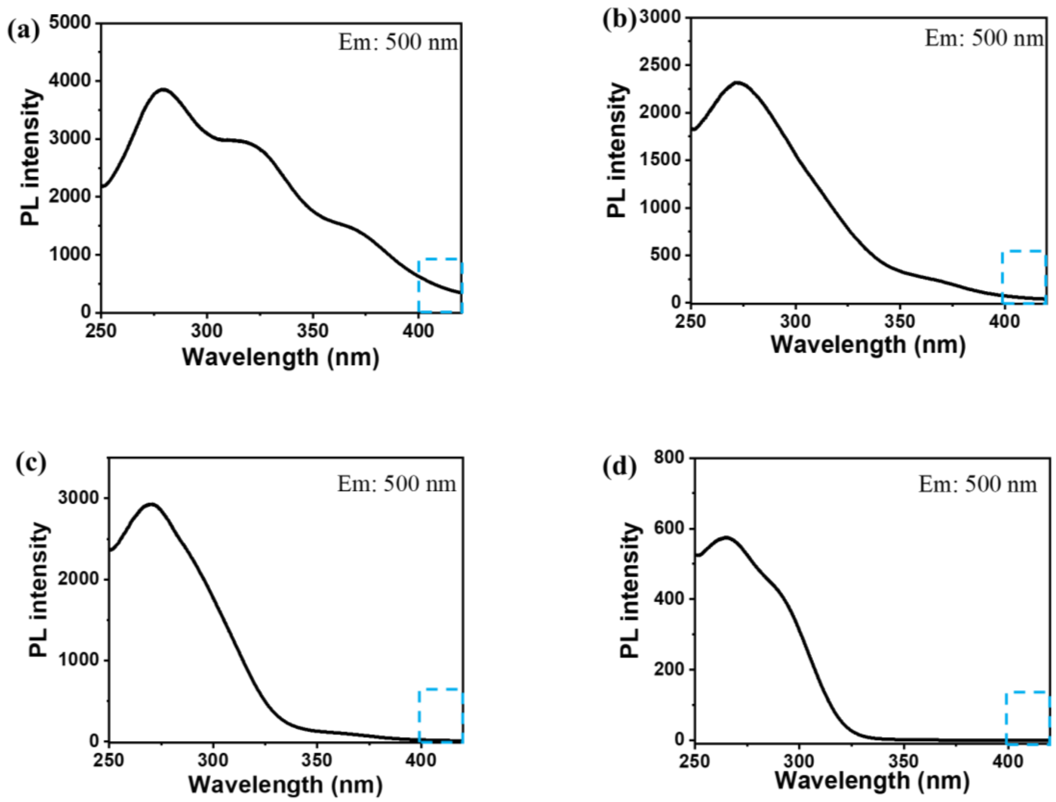

Supplementary Figure 14 Phosphorescence excitation spectra of samples with (a) 77% CBtCOONa; (b) 50% CBtCOONa; (c) 20% CBtCOONa; (d) 4% CBtCOONa (Em = 500 nm).

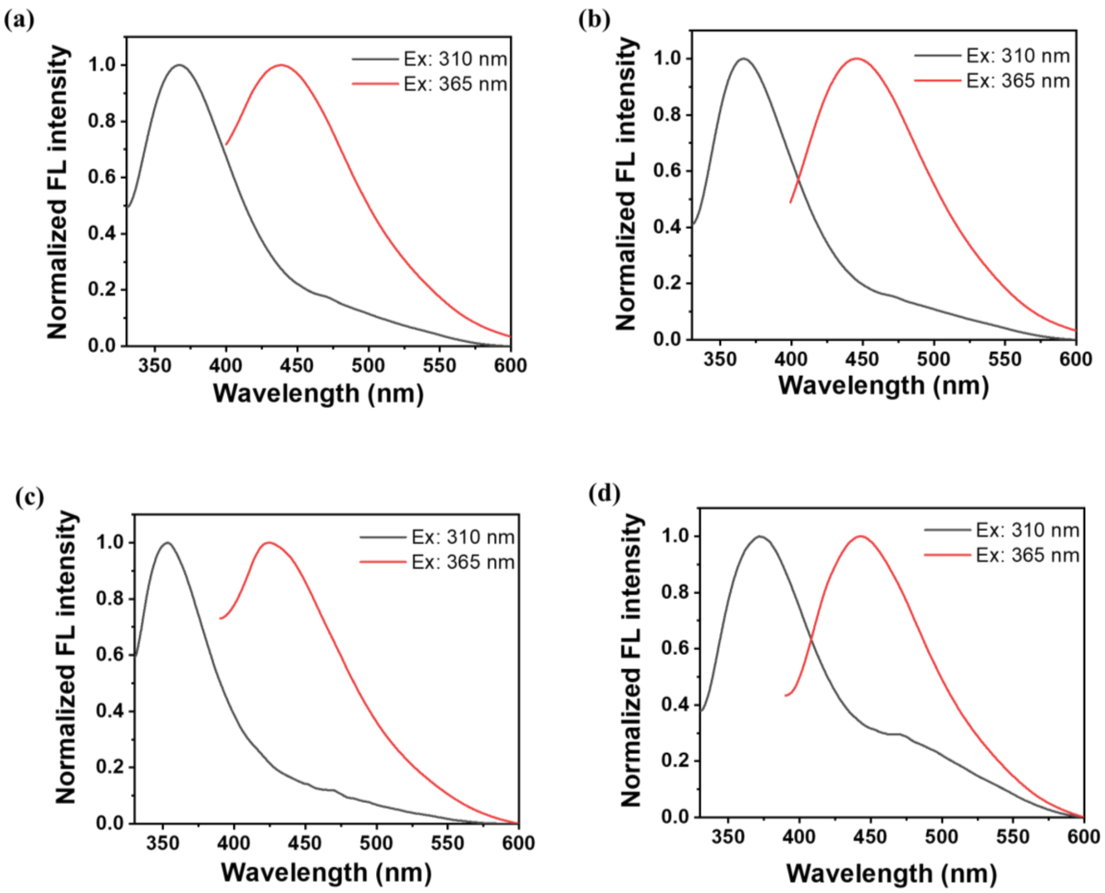

Supplementary Figure 15 Fluorescence spectra of (a) CBtCOOAl; (b) CBtCOOZn; (c) CBtCOOIn and (d) CBtCOOLa (Ex = 310 nm and 365 nm).

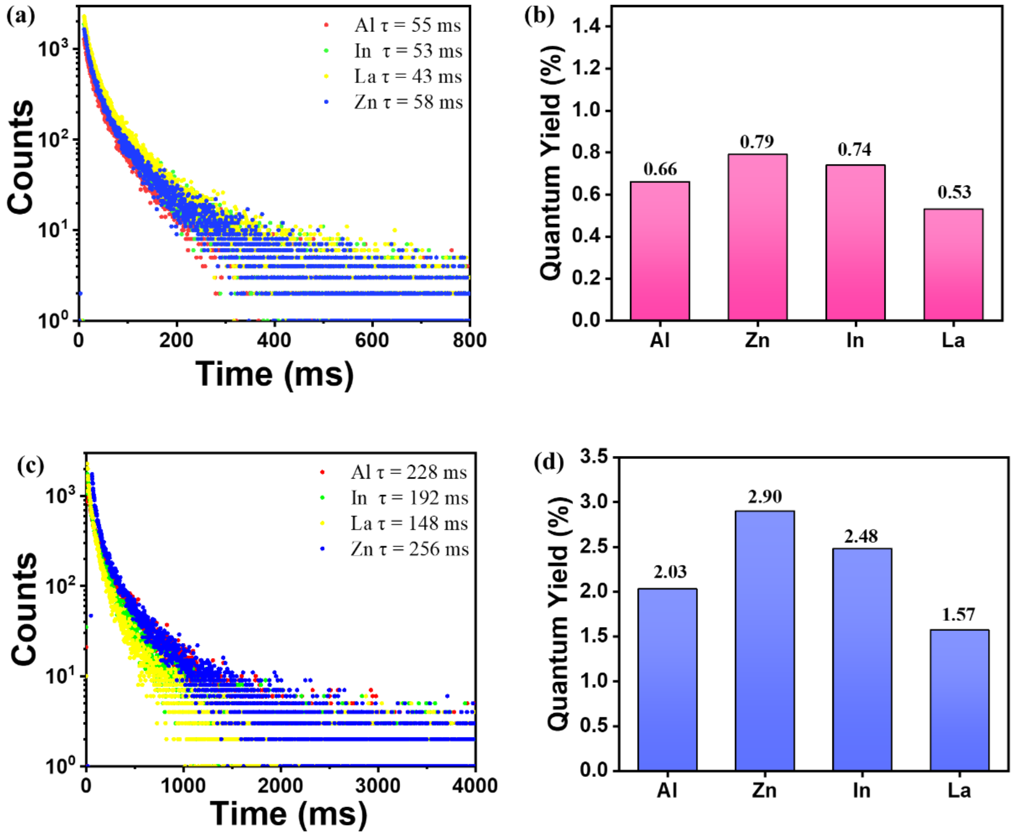

Supplementary Figure 16 (a) RTP lifetime spectra and (b) photoluminescence quantum yield of CBtCOOAl, CBtCOOZn, CBtCOOIn and CBtCOOLa (Ex = 310 nm, detection wavelength = 450 nm); (c) RTP lifetime spectra and (d) photoluminescence quantum yield of CBtCOOAl, CBtCOOZn, CBtCOOIn and CBtCOOLa (Ex = 370 nm, detection wavelength = 500 nm).

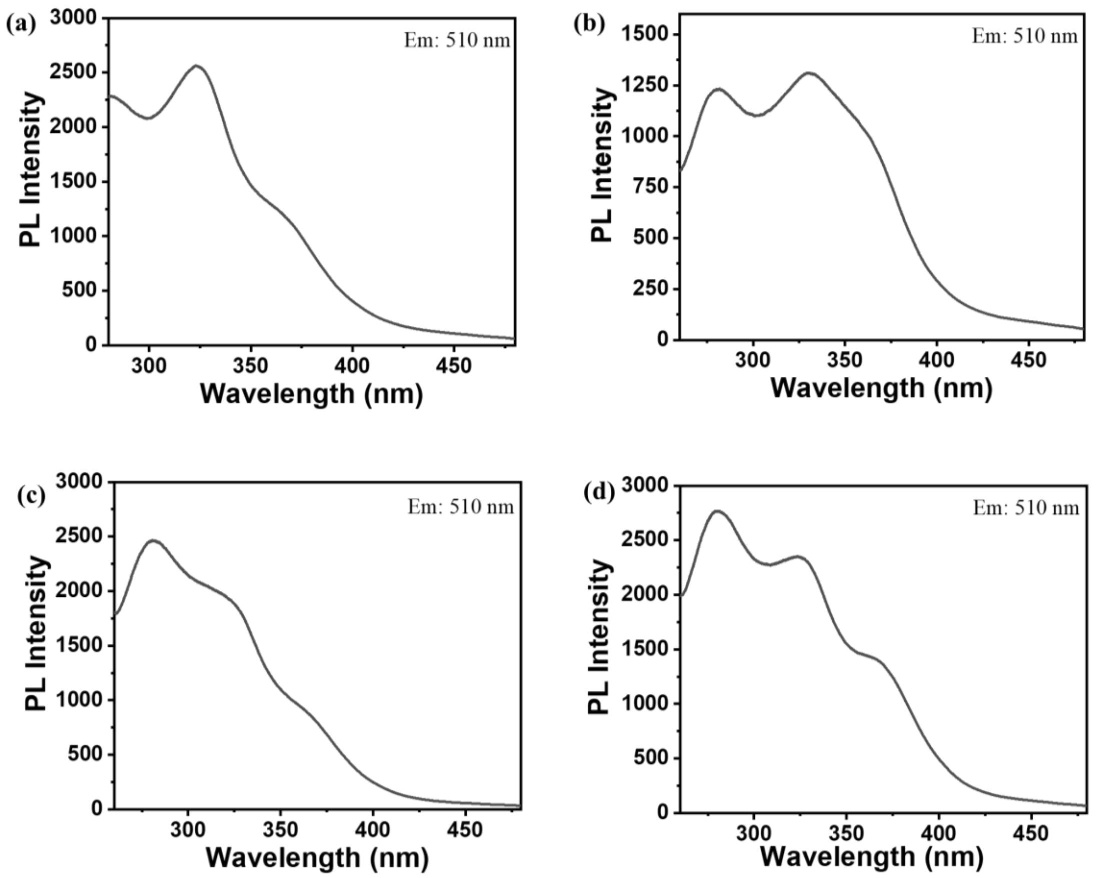

Supplementary Figure 17 Phosphorescence excitation spectra of (a) CBtCOOAl; (b) CBtCOOZn; (c) CBtCOOIn and (d) CBtCOOLa (Em = 510 nm).

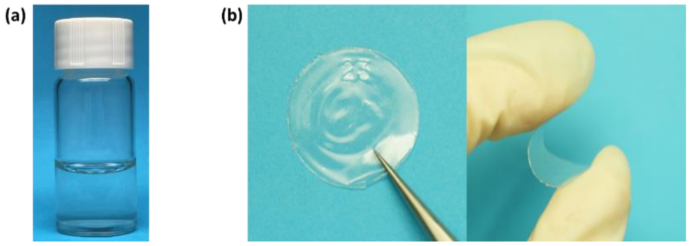

Supplementary Figure 18 (a) Photograph of CBtCOONa aqueous solution with 4 wt% concentration; (b) Photographs of CBtCOONa film.

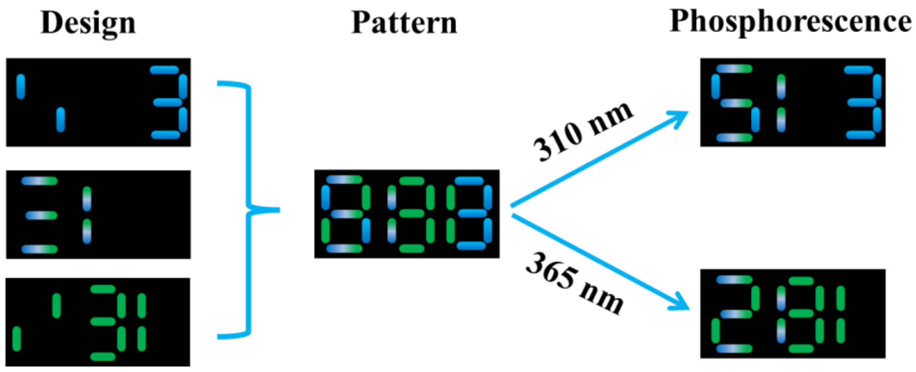

Supplementary Figure 19 Design schematic for a complex RTP pattern.

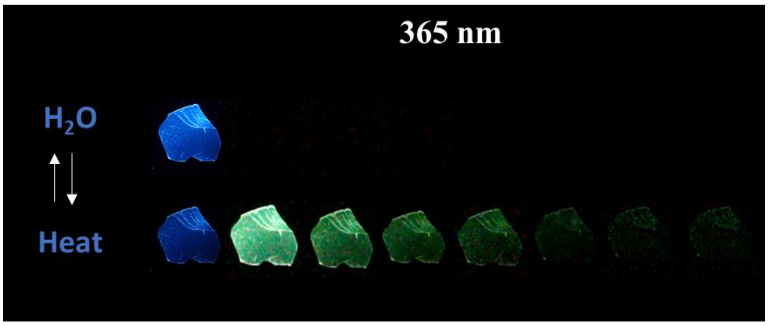

Supplementary Figure 20 Photographs of the reversible heating/water responsiveness process of CBtCOONa.
